# MicroRNAs and Long Non-Coding RNAs in Adrenocortical Carcinoma

**DOI:** 10.3390/cells11142234

**Published:** 2022-07-18

**Authors:** Mario Detomas, Claudia Pivonello, Bianca Pellegrini, Laura-Sophie Landwehr, Silviu Sbiera, Rosario Pivonello, Cristina L. Ronchi, Annamaria Colao, Barbara Altieri, Maria Cristina De Martino

**Affiliations:** 1 Department of Internal Medicine I, Division of Endocrinology and Diabetes, University Hospital Würzburg, University of Würzburg, 97080 Würzburg, Germany; detomas_m@ukw.de (M.D.); landwehr_l@ukw.de (L.-S.L.); sbiera_s@ukw.de (S.S.); c.l.ronchi@bham.ac.uk (C.L.R.); altieri_b@ukw.de (B.A.); 2 Dipartimento di Medicina Clinica e Chirurgia, Università “Federico II” di Napoli, 80131 Naples, Italy; cpivonello@gmail.com (C.P.); bianca.pellegrini02@gmail.com (B.P.); rosario.pivonello@unina.it (R.P.); colao@unina.it (A.C.); 3 Unesco Chair for Health Education and Sustainable Development, Federico II University, 80131 Naples, Italy; 4 Institute of Metabolism and System Research, University of Birmingham, Birmingham B15 2TT, UK; 5 Centre for Endocrinology, Diabetes and Metabolism (CEDAM), Birmingham Health Partners, Birmingham B15 2TT, UK

**Keywords:** miRNA, lncRNA, adrenocortical tumor, ACC, adrenocortical adenoma, prognostic markers

## Abstract

Non-coding RNAs (ncRNAs) are a type of genetic material that do not encode proteins but regulate the gene expression at an epigenetic level, such as microRNAs (miRNAs) and long non-coding RNAs (lncRNAs). The role played by ncRNAs in many physiological and pathological processes has gained attention during the last few decades, as they might be useful in the diagnosis, treatment and management of several human disorders, including endocrine and oncological diseases. Adrenocortical carcinoma (ACC) is a rare and aggressive endocrine cancer, still characterized by high mortality and morbidity due to both endocrine and oncological complications. Despite the rarity of this disease, recently, the role of ncRNA has been quite extensively evaluated in ACC. In order to better explore the role of the ncRNA in human ACC, this review summarizes the current knowledge on ncRNA dysregulation in ACC and its potential role in the diagnosis, treatment, and management of this tumor.

## 1. Introduction

Adrenocortical carcinoma (ACC) is a rare but aggressive form of adrenal tumor, with an annual incidence of 0.7–2.0 cases per million per year [1,2]. ACC is more common in women than in men (ratio 1.5) and although the incidence is higher at 40–50 years, the tumor can appear at any age [1,2]. Although ACC is associated with signs and symptoms of hormone excess in more than half of the cases (mostly caused by steroids, such as cortisol and androgens), in 10% of the patients, it may develop silently [2]. Because of its rarity, the diagnosis of ACC can be challenging. The new WHO histopathological classification suggests the use of multiparameter diagnostic algorithms, including classical pathological features, such as the Ki-67 proliferative index, together with multifactorial risk assessment systems, such as the Weiss score, reticulin algorithm, Lin–Weiss–Bisceglia and Helsinki system [3,4]. Additional immunohistochemical markers have been also described for differentiating the malignant etiology of adrenal masses [5,6,7,8].

Currently, the only curative treatment for ACC is a complete tumor resection [1]. However, often the tumor is diagnosed in the advanced stages where the curative approaches are still limited [2]. The only drug approved for the treatment of advanced ACC is mitotane [2,9], which has an adrenolytic effect and inhibits steroidogenesis [10]. Unfortunately, mitotane is not always effective and it is associated with multiple side effects [9,11,12]. Platinum-based chemotherapy or streptozotocin represent the first line of cytotoxic treatment in advanced ACC [13]. However, due to the limited efficacy, other chemotherapies or targeted therapies may also be considered in patients with ACC [2]. The prognosis of ACC is stage dependent with an overall 5-year survival rate, ranging from about 84% for European Network for the Study of Adrenal Tumors (ENSAT) tumor stage I to 15% for tumors with ENSAT stage IV [14]. In addition to the ENSAT stage, the prognosis of ACC may be influenced by other factors, such as age [15], tumor resection status [16], cortisol secretion [17], Ki-67 index [18] and molecular markers [19,20].

In the last few decades, genetic alterations in *CTNNB1*, *TP53*, *RB1* and *MEN1* genes have been associated with ACC [21,22]. Furthermore, several studies in different types of cancers (including ACC) show how the non-coding genetic materials, particularly non-coding RNAs (ncRNAs), have an important role also in the pathophysiology of the disease [23,24]. NcRNAs are RNA molecules that do not encode proteins but regulate the gene expression at an epigenetic level. Two examples of ncRNAs are the microRNAs (miRNAs) and long non-coding RNAs (lncRNAs). The major differences between miRNAs and lncRNAs are related to their size and role. MiRNAs are about 20–25 nucleotides long, while lncRNAs are more than 200 nucleotides.

In the human genome, about 2300 miRNAs have been identified [25]. Some of them are expressed in many or all human tissues and some others are tissue specific. Generally, as epigenetic mediators, miRNAs, upon binding to the 3′ or 5′ untranslated regions (3′UTR or 5′UTR), gene promoter region or coding sequence, negatively regulate the protein levels of the target messenger RNA (mRNA) through either translational inhibition or transcript degradation [26,27]. Recent evidence indicates that miRNAs can function as oncogenes (colloquially known as “oncomirs”) that regulate cancer-related processes, such as cell growth, cell proliferation and the apoptotic pathway [28].

In the adrenal gland, miRNAs have been found to play an important role not only at the physiological level, but also in the onset and development of both adrenocortical adenomas (ACA) and ACC. MiRNAs are found both intratumorally and in circulation. Since circulating miRNAs are easily detectable and highly stable, they are promising diagnostic, prognostic, and therapeutic biomarkers in several tumors, including ACC [23,24,29].

While miRNAs are mostly epigenetic regulators that act post-transcriptionally, lncRNAs exhibit an important role at the transcriptional and post-transcriptional level [30,31]. LncRNAs can be either linear or circular RNA molecules. Linear lncRNAs have a regulatory role at the transcriptional and post-transcriptional level [23,32], while circular lncRNAs are also involved in splicing, transcription, and can inhibit ribosomal maturation [33]. Furthermore, they are able to regulate miRNA’s function, preventing or inducing mRNA degradation [34].

In the current review, we present a summary of the studies in which the expression and role of miRNAs and lncRNAs were investigated in ACC and other tumor entities. Additionally, we focus on the miRNA and lncRNAs used for the diagnosis (especially in the differentiation between malignant and benign tumors) and for establishing the prognosis of ACC.

## 2. Circulating miRNA in Adrenocortical Carcinoma

As mentioned above, miRNAs are not only present intratumorally, but they can be found also in blood and in other body fluids, such as urine, sperm, breast milk and saliva [35]. Intracellular miRNAs can be released in plasma both actively or passively (i.e., in the case of necrosis). Actively secreted miRNAs are either incorporated in extracellular vesicles (EV) or in macromolecular complexes (such as high density lipoproteins) [35] and exhibit a role also in cell communication, by transferring epigenetic material between different cells.

As miRNAs can be easily extracted from different biological fluids, they have tissue- or cell-type specificity and may vary according to the disease progression. For these reasons, they have been used as biomarkers in several studies throughout the last few decades to differentiate between different cancer stages and even for the measurement of therapy responsiveness [36]. Certain miRNAs are found to be increased in the plasma of patients with ACA or ACC. For these reasons, the aim of several studies was to identify a specific subset of miRNAs for ACA or ACC that could facilitate the differentiation between these two entities in a non-invasive way, since currently non-invasive differentiation is mostly performed with imaging technologies [1].

Several studies have already reported that miRNAs can be used in the differentiation among ACA, ACC, and normal adrenal glands (NAG) (Table 1) [37,38,39,40,41,42,43].

Chabre et al. [37] performed one of the first studies investigating the levels of the circulating miRNAs to assess their predictive diagnostic and prognostic role in ACC. The authors performed a microarray analysis on the serum of 56 patients, 14 patients with ACA, 9 with non-aggressive ACC, 14 with aggressive ACC (recurring tumors or tumors that were already metastatic at diagnosis) and 19 healthy controls were included. Specifically, five miRNAs appeared to be dysregulated. With respect to ACA and the healthy controls, miR-483-5p was found to be present only in the serum of the aggressive ACC patients. These findings were partially confirmed in further studies, although the miR-483-5p was found to be overexpressed in less aggressive ACC [38,39,40,41,43]. The other important findings of Chabre et al. [37] were related to miR-195 and miR-335 and were identified to be significantly decreased in both tumor tissue and serum samples of ACC, compared to ACA. Furthermore, the low levels of miR-195 also correlated with a bad prognosis for ACC patients.

Another study investigated by RT-qPCR the levels of five miRNAs in circulation, which had previously been shown to be dysregulated in cancer and selected from miRNA profiling studies in ACC (miR-let-7d, miR-34a, miR-195, miR-214, and miR-483-5p). This study identified higher levels of miR-34a and miR-483-5p in the serum of ACC patients compared to ACA patients [41]. Moreover, although the area under the ROC curve of both miRNAs revealed them to be good diagnostic biomarkers, miR-34a and miR-483-5p at circulating levels do not correlate with the extent of disease, disease-free survival or position emission tomography scan avidity (tumor SUV). Interestingly, miR-34a was not previously reported to be overexpressed in ACC tissues, whereas its circulating levels are high in ACC patients. This was explained by the active secretion of miR-34a from tumor cells that consequently lead to a depletion at the intracellular level.

Another study investigating 12 ACA and 13 ACC patients [38] reported higher levels of miR-100, miR-181b, miR-184, miR-210, miR-483-5p in the unfractionated plasma of ACC patients in comparison to ACA. The authors found that a combination of miR-210 and miR-181b, as well as the ratio miR-100/miR-181b, had the highest diagnostic accuracy values to differentiate ACA from ACC, with a sensitivity of 89% and 78%, and a specificity of 75% and 100% respectively. In a more recent study performed by the same group, the authors showed that levels of miR-483-5p in circulation had a similar sensitivity and specificity (87% and 78%, respectively) in the differential diagnosis between ACA and ACC [43]. In addition to these, the same group evaluated the EV-associated microRNAs (because of their high specificity) as potential diagnostic molecular markers in ACC patients [40]. Taking into consideration a cohort of 24 ACA and 22 ACC patients, two EV-associated miRNAs that were higher in ACC compared to ACA were identified, including miR-101 and once again miR-483-5p. The EV-associated miR-101 showed a sensitivity of 69% and a specificity of 83% in the differential diagnosis between ACC and ACA, whereas the EV-associated miR-483-5p presented the highest diagnostic accuracy with a sensitivity of 87% and a specificity of 94%. Another important finding was the higher level of miR-363-3p and miR-451a in adrenal myelolipomas (AML) compared to both ACC and ACA [42]. However, the miR-483-5p analyzed in the urine did not show any difference between ACC and ACA patients [43].

Circulating miRNAs that are more underexpressed or overexpressed in ACC than in NAG or adrenal adenoma are shown in Table 1.

## 3. Tumorigenesis Pathways in Which Five of the Main ACC Circulating miRNAs Are Involved

### 3.1. miR-100

miR-100 exerts antitumor activity by regulating the CXC chemokine receptor type 7 (CXCR7) [44]. CXCR7 is a chemokine receptor that can induce tumor progression by activating the MAPK signaling pathway. In ACC, CXCR7 is found to be overexpressed in local tumors and metastases, suggesting that some patients could benefit from a CXCR7-targeted therapy [45].

miR-100 has been found to exert an antitumoral function in other cancers, including hepatocellular carcinoma (HCC) [44,46] and nasopharyngeal carcinoma [47]. In the case of HCC, miR-100 downregulation leads to an overexpression of the insulin growth factor 2 (IGF2), which has oncogenic activity in several types of cancers. Interestingly, IGF2 and IGF1 receptors (IGF1R) are frequently overexpressed in ACC [2,48]. This leads to an auto-paracrine loop, where IGF2 activates IGF-downstream pathways, which promote cell proliferation, motility and prevent cell apoptosis [49,50].

One of the miR-100 targets is the polo-like kinase 1 (Plk1) mRNA, which is a critical mitotic regulator at the G2/M transition [47].

Presently, whether the miR-100 could play a role in the pathogenesis of ACC via Plk1 or CXCR7 downstream pathways has not yet been clarified; on the contrary, in vitro studies have pointed out a direct action of miR-100 on the expression of IGF1R and mammalian target of rapamycin (mTOR) [51].

### 3.2. miR-181b

B-cell lymphoma 2 (Bcl-2), which is an important regulator of apoptosis, is one of the targets of miR-181b associated with tumorigenesis. Bcl-2 was reported to suppress the growth of ACC SW-13 cells in vitro [52]. Another target of miR-181b is the ataxia telangiectasia mutated (ATM) transcript, which is a key activator of the DNA-damage response to double-strand breaks. In ACC, a loss of function of ATM is observed in 4% of cases [22,53].

miR-181b plays an essential role in the regulation of angiogenesis in different types of tumors [54,55]. However, whether miR-181b modulation could exert an antiangiogenic and pro-apoptotic action in ACC remains to be clarified.

### 3.3. miR-195

miR-195 is implicated in several types of cancers. In HCC, miR-195 plays a pathological role by targeting cyclin D1, cyclin-dependent kinase 6 (CDK6) [56]. CDK6 is regulated by cyclins, more specifically by cyclin D proteins, and is fundamental for the G1 phase progression and G1/S transition of the cell cycle. In ACC, it has been shown that high levels of CDK6 are associated with a shorter time to relapse and a poorer survival of patients [57].

Currently, whether miR-195 exerts its action via CDK6 or ARL2 in ACC remains to be clarified. However, in the meantime, miR-195 has been subjected to in vitro studies on ACC human cells, identifying its specific role involving DICER and TARBP2 [58].

### 3.4. miR-483-5p

miR-483-5p is involved in the development of various cancers. It is well known that one of its targets is MAPK interacting serine/threonine kinase 1 (MKNK1), which inhibits Wilm’s tumor cell proliferation and induces apoptosis [59]. Furthermore, it has been shown that miR-483-5p facilitates esophageal cancer proliferation, migration, and invasion by silencing potassium voltage-gated channel subfamily Q member 1 (*KCNQ1)* [60]. *KCNQ1* is a gene that encodes a potassium channel protein present in various tissues.

A similar role of miR-483-5p could be hypothesized also in ACC thanks to in vitro evidence showing a correlation between miR-483-5p expression and cell proliferation in in vitro [61]. However, the underlying cellular mechanisms have not been elucidated yet.

## 4. Circulating miRNAs Associated with Hormonal Secretion in the Adrenal

Other circulating miRNAs have been associated with hormone-secreting adrenal tumors or hyperplasia. Cortisol-producing adenomas (CPA) and cortisol-secreting ACC were identified to have higher levels of three circulating vesicle-free miRNAs when compared with non-secreting adrenal adenomas, including miR-22-3p, miR-27a-3p and miR-320b [62]. Interestingly, a significant correlation between the 24h-urinary free cortisol levels and the expression of these three miRNAs was identified. Furthermore, Igaz et al. proved that the expression of miR-27a is upregulated by dexamethasone, whereas it is suppressed by adrenocorticotropin [63]. These results suggest an influence of cortisol in the expression of some miRNAs.

Another circulating miRNA, which has been identified in the context of hypercortisolism, is miR-182-5p and has been proposed as a biomarker for Cushing’s disease (CD) [64]. The results from RNA-sequencing (RNA-seq) suggested miR-146b-5p as a possible biomarker of CPA; however, the results of qPCR did not confirm it [64].

Interestingly, the circulating levels of miR-133a-3p and miR-200b-3p, specifically secreted by muscle tissue, and therefore well known as myomiRs, are higher in patients with CD and Cushing’s syndrome (CS) compared to healthy subjects. These miRNAs, correlating with the 24h urinary free cortisol levels, could represent promising markers of hypercortisolism [65].

In consideration of primary hyperaldosteronism, a study revealed the correlation of three miRNAs (miR-30e-5p, miR-30d-5p, and miR-7-5p) with adrenal hyperplasia regarding aldosterone producing adenomas (APA) [66]. Although higher levels were observed in bilateral adrenal hyperplasia in the context of hyperaldosteronism, the analysis of these three miRNAs did not show high diagnostic accuracy, since the sensitivity and specificity were not over 62% for all the considered miRNAs [66]. Furthermore, some miRNAs have been detected to differ between CD and ectopic CS patients. In a recent publication, it was reported that miR-16-5p, miR-145-5p and miR-7g-5p vary between the two groups, and could therefore represent promising biomarkers for the differentiation of ACTH-dependent CS [67].

Circulating miRNAs whose expression is related to hormonal secretion in adrenal tumors are shown in Table 1.

## 5. Tissue miRNAs in Adrenocortical Carcinoma

Similar to miRNAs circulating in plasma and other body fluids of patients, identifying specific miRNAs is also promising in adrenal tumors. The techniques used for the initial analyses were microarray, TaqMan low density arrays (TLDA) and RT-qPCR. The first study was performed with microarray expression assays on 36 tissue samples, including 10 NAG, 10 endocrine inactive adenomas (EIA), 9 CPA and 7 ACC [68]. Tömbol et al. performed an analysis with TLDA and reported higher expression levels of miR-184 and miR-503 in ACC in comparison with NAG, EIA and CPA, while miR-210 expression was significantly higher in ACC compared to the CPA. On the other hand, the expression of miR-511 and miR-214 was significantly lower in ACC compared to the other groups; and the expression of miR-375 was significantly higher in NAG than ACC and CPA.

As identified in the circulation, miR-483-5p was found to be overexpressed also in ACC tissue [69]. The microarray analysis also revealed overexpression of miR-93, miR-106b, miR-130b, miR-135a, miR-143, miR-148b, miR-181b, miR-320a, miR-450a, miR-503, miR-542-3p, miR-542-5p in ACC when compared to ACA [69].

The upregulation of the miR-483-5p and miR-503 in ACC was identified also in other studies, some of which use RNA-seq [21,37,61,70,71,72,73]. Compared to the microarray analysis, RNA-seq allows identification of more, previously not known, miRNAs. One example is miR-508-3p that was identified to be upregulated in ACC tissue in more recent studies [21,73]. Other important miRNAs that are often reported to be overexpressed in ACC are miR-483-3p that together with miR-483-5p represent the two mature forms of miR-483 [21,61,70,72] and miR-210 [37,68,70].

Moreover, several miRNAs were also less expressed in ACC compared to ACA and NAG. In 2009, by using microarray, a low expression of miR-195 and miR-335 was identified in ACC compared to ACA and to NAG [69]. The underexpression of miR-195 in ACC was confirmed also in several further studies with microarray analyses [37,61], including RT-qPCR [72] and RNA-seq [21], while low expression of miR-335 was confirmed only with TLDA [74].

In 2011, a study reported lower levels of miR-497 in ACC in comparison to ACA and NAG. This was confirmed also in three more recent analyses using microarray [37,75] and RNA-seq [21].

A significant difference in the expression of miR-7 in ACC, ACA and NAG was also reported [69]. In fact, miR-7 was identified to be significantly overexpressed in NAG compared to the two counterparts. This finding, however, was not confirmed in more recent investigation studies regarding miRNA differential expression between ACC and NAG.

Tissue MiRNAs that are more underexpressed or overexpressed in ACC than in NAG or adrenal adenoma are shown in Table 2.

## 6. Tumorigenesis Pathways in Which Principal ACC Tissue miRNAs Are Involved

### 6.1. miR-184

A target of miR-184 is IGF1R. As reported above, the IGF pathway is over-activated in ACC. Furthermore, the overexpression of IGF1R has been described to be involved in several types of cancers, i.e. prostate cancer [76]. On the other hand, an additional target of miR-184, programmed cell death 4 (PDCD4), has been described to be a tumor suppressor by acting at the level of c-Myc and Bcl-2, inhibiting the cell proliferation and survival in nasopharyngeal cancer cells [77]. As reported above, it has been demonstrated that Bcl-2 suppressed tumor proliferation in vitro in the ACC SW-13 cells [52].

### 6.2. miR-375

miR-375 has been shown to be involved in several types of cancers, such as ovarian cancer [78], HCC [79], esophageal [80] and colorectal cancer [81]. Particularly, in colorectal cancer, it has been shown that *PIK3CA* is a target gene of miR-375 [81]. The *PIK3CA* amplification correlated with PI3K/Akt signaling pathway activity, which modulates cell proliferation through several downstream targets, including mTOR, p21 and p27. A dysregulation of the mTOR pathway was reported in a subset of ACC. Although in vitro studies presented promising results, only a few clinical studies about mTOR inhibitors in ACC patients have been reported [2,82,83,84,85,86,87].

In ovarian cancer, it has been shown that miR-375 targets pair box gene 2 (PAX2), which plays a fundamental role in the development and proliferation of several cell lines [78].

Furthermore, miR-375 was identified to be overexpressed in HCC cell lines resistant to everolimus. Interestingly, these everolimus resistant cells were also characterized by an overexpression of c-Myc [88]. c-Myc is well known to be overexpressed in the cytoplasm of ACC cells [89] and its modulation has been shown to induce the responsiveness to paclitaxel in ACC cell lines [90].

Nowadays, is not clear whether PAX2, PI3K/Akt/mTOR or c-Myc represent the main targets of miR-375, but in vitro studies suggested a role of miR-375 in the pathogenesis of ACC through the regulation of PI3K/Akt/mTOR [91].

### 6.3. miR-483-3p

The expression of miR-483-3p is up-regulated by IGF2 and transcriptionally induced by β-catenin [92,93]. The Wnt/β-catenin signaling pathway plays a major role in the development of the adrenal glands. Somatic activating mutations in the *CTNNB1* gene encoding β-catenin are recognized in about 40% of ACC [19,21,73].

miR-483-3p is overexpressed in many types of cancers, such as hepatocellular carcinoma [92], colorectal cancer and anaplastic thyroid carcinoma [94]. MiR-483-3p is known to target BBC3/PUMA in HCC and colorectal cancer. PUMA is a downstream target of p53, which is an antagonist of anti-apoptotic Bcl-2 family proteins, and consequently induces apoptosis [95]. In anaplastic thyroid cancer, it is known that overexpression of miR-483-3p targets partitioning-defective 3 (PARD3) [94], which regulates cell polarity, cell migration and cell division.

In conclusion, miR-483-3p is involved in the tumorigenesis of several cancers, as it induces proliferation and migration by regulating the PARD3 pathway and inhibits apoptosis via the Bcl-2 pathway. miR-483-3p has been associated with Wnt/β-catenin signaling pathway alterations that are quite frequent in ACC. Furthermore, in vitro studies suggest an anti-apoptotic role of miR-483 in the NCI-H295R ACC cell line via targeting the BBC3/PUMA, but whether this effect is mediated by Bcl-2 or PARD3 in ACC remains to be clarified [61].

### 6.4. miR-503

The primary transcript of miR-503 is excised into two different forms of mature miRNA, miR-503-5p and miR-503-3p. MiRNA-503-5p regulates cell proliferation, migration and invasion by using targets such as IGF1R, Bcl-2, PI3K [96,97]. On the other hand, miR-503-3p induces lung cancer cell apoptosis by regulating CDK4 and p21 [98]. CDK4 is an enzyme that, as is the case with CDK6, has an important role in the cell cycle, especially for the G1/S transition. Copy number gains or amplification in the CDK4 oncogene locus are prevalent in ACC between 7% and 40% [2,21,53,73]. P21, also known as cyclin-dependent kinase inhibitor, is the major target of p53 involved in the cell cycle arrest.

Presently, whether miR-503 plays a role via IGF1R, Bcl-2 and PI3K or CDK4 in ACC remains to be clarified; however, in vitro studies suggest a potential role of miR-503 on ACC human cell proliferation.

## 7. Tissue-miRNAs Associated with Hormonal Secretion

Different miRNAs are involved in the pathogenesis of primary hyperaldosteronism due to APA. Examples are miR-23, miR-34a and miR320a-3p, which are overexpressed in APA [99,100,101]. The target of miR-23 and miR-34a is TWIK-related acid-sensitive K channel 2 (*TASK-2*), a gene that regulates the potassium permeability in aldosterone-producing cells. The downregulation of this gene is involved in the overexpression of steroidogenic enzymes, such as *STAR* and *CYP11B2* [100]. MiR-320a-3p is a regulator of both *CYP17A1* and *CYP11A1,* which are important regulators of the steroidogenesis [101].

Not only the overexpression, but also the underexpression of miRNAs is related to primary hyperaldosteronism. The low expression of miR-193a-3p, miR-125a-5p and miR-125b-5p in APA associated with the regulation of aldosterone hypersecretion [101,102], while miR-10b and miR-24 have been identified to play a role in both aldosterone and cortisol hypersecretion [103,104].

In the context of primary pigmented nodular adrenocortical disease, which is a bilateral adrenal hyperplasia associated with the Carney complex and primary hypercortisolism, the overexpression of miR-449 was identified [105]. Recently, unsupervised methylome classification identified specific miRNA groups associated with hormonal secretion, i.e., mi2 (including miR-100-5p) with aldosterone and mi5 (including miR-494-3p) with cortisol secretion [105,106].

Tissue miRNAs whose expression is related to hormonal secretion in adrenal tumors are shown in Table 2.

## 8. Specific Role of miRs in the Pathogenesis of ACC

Some in vitro studies in human ACC primary cultures or human ACC cell lines have supported the specific role of some miRNAs in the pathogenesis of ACC by targeting some cellular pathways involved in cellular proliferation and survival.

### 8.1. miR-7

miR-7 has been found to be underexpressed in ACC tissue [23,69]. In vitro studies in human ACC cell lines and in human ACC primary cultures suggest that the restoration of miR-7 levels can lead to G1-cell cycle arrest. These results were supported by in vivo studies in ACC xenograft models, demonstrating an anti-tumoral effect of miR-7 restoration via the RAF1 and mTOR level reduction and CDK1 inhibition [107].

### 8.2. miR-200b

In in vitro studies performed on the NCI-H295 cell line, miR-200b reduced the expression of MATR3, a nuclear protein involved in the regulation of transcription and in the PKA signaling pathway, potentially contributing to carcinogenesis [108].

### 8.3. miR-203

Albeit no evidence is actually available regarding miR-203 expression in human ACC samples, miR-203 was found to be overexpressed in APA. Additionally, in human ACC cell line HAC15, inhibition of this miRNA has been related to an increase in cell proliferation and aldosterone secretion via the regulation of WNT5, of CYP11B2 mRNA expression [109].

### 8.4. miR-193a-3p

Intratumoral underexpression of miR-193a-3p has been suggested to be related to the oversecretion of aldosterone from an adrenal adenoma, while no data are available on the tissue level of this miRNA in ACC. However, it has been observed that miR-193a-3p can inhibit the in vitro proliferation in the NCI-H295 cell line, inducing G1 phase arrest and early apoptosis mediated by an overexpression of BAX proteins and an underexpression of BCL-2 proteins. Moreover, it can reduce the secretion of aldosterone by downregulating the expression of CYP11B2 mRNA [102].

### 8.5. miR-483-5p and miR-483-3p

Underexpressed tissue miR-483-5p and miR-483-3p and lower circulating miR-483-5p levels can be found in ACC. Their possible role in the pathogenesis of ACC has been confirmed in NCI-H295 cells, in which a downregulation of both miR-483-3p and miR-483-5p leads to reduced proliferation in vitro, while only a downregulation of miR-483-3p increases apoptosis. In particular, a pro-proliferation and anti-apoptotic action of miR-483-3p was achieved by the silencing p53 upregulated modulator of apoptosis (PUMA) [61]. miR-483-5p has been found to be upregulated in ACC, and associated with the advanced clinical stage, as compared to ACA or NAG and appears as a potential biomarker for the diagnosis and prognosis of ACC. The N-Myc downstream-regulated gene (NDRG) family member NDRG2 was the most downregulated target gene of miR-483-5p. Inhibition of miR-483-5p or restoration of NDRG2 effectively suppressed the invasive potential of H295R and SW13 cells, suggesting that miR-483-5p acts as oncomiR in ACC pathogenesis [110].

### 8.6. miR-139-5p

As with miR-483-5p, miR-139-5p has been found to be upregulated in ACC as compared to ACA or NAG and appears as a potential biomarker for diagnosis and prognosis [110]. miR-139-5p expressions have been demonstrated to be inversely correlated with the N-Myc downstream-regulated gene (NDRG) family member NDRG4 expressions in ACC, and in ACC cell model inhibition of miR-139-5p or restoration of NDRG4, which effectively suppressed the invasive potential of H295R and SW13 in vitro. Thus, as for miR-483-5p, miR-139-5p might also act as oncomiRNAs in ACC pathogenesis.

### 8.7. miR-205

miR-205 was found to be lower expressed in ACC tissue compared to ACA [23,52]. In vitro studies in SW-13 described that miR-205 exerts a pro-apoptotic action by reducing the expression of BCL-2 [52].

### 8.8. miR-375

In in vitro studies in NCI-H295 cells, miR-375 was found to modulate cell proliferation by targeting the MTDH/Akt pathway [91].

### 8.9. miR-100 and miR-99

miR-100 and miR-99 were found to be underexpressed in childhood adrenocortical tumors (ACT) compared to adult ACC. In in vitro studies, in pediatric ACT primary cultures, the underexpression of miR-100 induces the overexpression of IGF1R and mTOR. In both NCI-H295 and SW-13 cell lines, the miR-100 effect was confirmed and it was found to be dose dependent [51].

### 8.10. miR-195 and miR-497

Intratumoral underexpression and lower levels of miR-195 in the circulation of ACC patients, as well as intratumoral underexpression of miR, have been reported. To explore the role of these miRNAs in the pathogenesis of ACC, H295 cells have been exposed to high levels of miR-195 and miR-497 mimics. This exposure induced a decrease in DICER and TARBP2 mRNA and protein expression, resulting in cell proliferation inhibition and cell apoptosis induction. These data suggest that DICER and TARBP2 miRNA might have an oncogenic role in ACC. In contrast, their role as onco-suppressors has been described in other cancers [58]. The in vitro dysregulation of other miRNAs, such as miR-503, miR-210, miR-181b, miR-184, has also been correlated with abnormal proliferation in ACC cells, but the molecular targets involved in this dysregulation has not been clarified yet [61].

**Figure 1:**
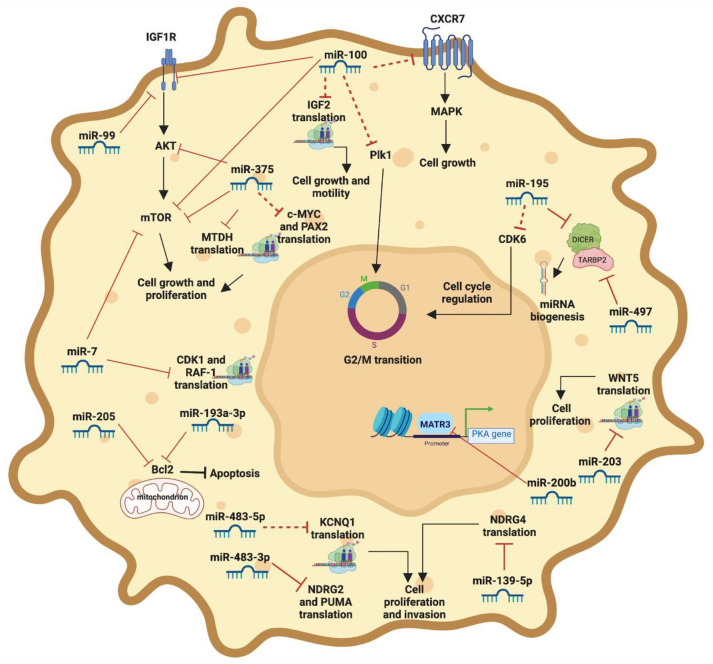
The image depicts the tumorigenesis pathways in which the principal miRNAs are involved in ACC pathogenesis. Dotted red lines represent the potential pathways regulated by miRNAs evaluated in other types of cancer. Continuous red lines represent the pathways regulated by miRNAs evaluated in ACC models. Image created with BioRender.com.

The main role of miRNAs involved in the pathogenesis of ACC is shown in Figure 1.

## 9. Role of miRNA in the Prognosis and Therapy of ACC

Currently, only a few miRNAs could be used as prognostic markers in patients with ACC. Chabre et al. [37] investigated circulating miRNA and identified low miR-195 and high miR-483-5p circulating levels as strong predictive/prognostic values for aggressive ACC. In a recent study performed on 48 ACC patients, the same group [29] showed that patients with lower postoperative miR-483-5p levels demonstrated a significant longer recurrence-free and overall survival rate than patients with higher miR-483-5p levels.

The association between intratumoral overexpression of miR-483-5p and bad prognosis was previously reported by another study [69]. Here, the upregulation of miR-483-5p and the downregulation of miR-195 in ACC patients was associated with poorer disease-specific survival.

Other miRNAs associated with shorter overall survival in ACC patients described in the literature are miR-503, miR-1202 and miR-1275 [61].

The most used chemotherapeutic regimen in patients with advanced ACC is the combination of etoposide, platinum compound and doxorubicin in association with mitotane [13].

In a recent study, miR-431 was found to be underexpressed in ACC patients with progressive disease [111]. In two ACC cells lines, the NCI-H295R and a primary ACC cell line, the upregulation of the miR-431 leads to the decrease in the half maximal inhibitory concentrations of mitotane and doxorubicin, together with an increase in cellular apoptosis [111].

To date, there is scant evidence regarding the potential use of miRNAs as prognostic markers of response to therapy in ACC. In gastric cancer cells, it has been reported that miR-503 modulates the cisplatin resistance by targeting *IGF1R* and *Bcl2* [96]. Additionally, in the last few years, the number of identified miRNAs involved in the process of cisplatin resistance or sensitivity has increased [112].

An in vivo study in a mouse NCI-H295R xenograft model showed that the levels of miR-483-5p decreased after the combined treatment with mitotane and 9-cis retinoic acid [113]. Furthermore, an in vitro study showed that the levels of miR-210 were increased after liposomal etoposide, doxorubicin, cisplatin, and mitotane treatment [114]. More studies are needed to evaluate the potential use of miRNAs as potential markers for the response to therapy.

## 10. Long Non-Coding RNAs in ACC

lncRNAs are non-protein-encoding molecules longer than miRNA, at least 200 nucleotides long. To date, more than 200,000 lncRNAs have been described in the human tissues.

Although there are not many studies present in the literature about lncRNA and ACC, the available data showed that analyses of lncRNAs could be important in the diagnosis and prognosis of ACC.

The first study performed on lncRNAs and ACC identified 956 lncRNAs differently expressed between ACC and NAG and 85 lncRNAs differently expressed between ACC and ACA [115]. In this study, performed with a microarray platform, 476 lncRNAs were upregulated and 480 were downregulated in ACC. Among the identified lncRNAs, some had an already known carcinogenic role, such as *H19*, growth specific arrest 5 (*GAS5*), metastasis-associated lung adenocarcinoma transcript 1 (*MALAT1*), and psoriasis-associated RNA induced by stress (*PRINS*) [115]. Particularly, *PRINS* is associated with metastatic ACC [115]. *PRINS* was first described to be overexpressed in epidermal cells of psoriatic patients [116]. In epidermal cells, *PRINS* regulates *G1P3*, an apoptotic gene [117]. Silencing *PRINS* has been shown to upregulate the Wnt/β-catenin pathway crucial for the pathogenicity of ACC [115]. The overexpression of *MALAT1* in ACC compared to the ACA was confirmed also by another study, suggesting a pathophysiological role of *MALAT1* in the development of ACC [118]. *MALAT1* has been associated with several other cancers (such as osteosarcoma and breast cancer) and functions as an oncogene or tumor suppressor gene [119,120].

More recently, data derived from RNA-seq showed that ACC could be differentiated from ACA using the lncRNA expression profile [121]. Particularly, this study demonstrated that the majority of lncRNAs were downregulated in ACC compared to ACA, whereas only a few lncRNA were significantly upregulated in ACC [121]. Among the upregulated lncRNAs, the colorectal neoplasia differentially expressed (*CRNDE*) is overexpressed in various human cancers [122] and has been shown to correlate with cell proliferation, migration and invasion, by regulating the Notch1 and the PI3K/AKT signaling pathway [123,124]. Both signaling pathways are considered to play a potential role in the pathogenesis of adrenocortical tumors [87,125]. However, different to other tumor types [122], *CRNDE* seems not to correlate with the prognosis in ACC patients [126].

Concerning circular ncRNAs, a recent study showed that circ-CCAC1 facilitates ACC cell proliferation, migration, and invasion by regulating miR-514a-5p [127]. In this study, circ-CCAC1 (which was demonstrated to act as an oncogene) was overexpressed in ACC tissue samples and cell lines and was also associated with a bad prognosis.

## 11. Conclusions

The role of epigenetic modifications in the pathogenesis of various kinds of tumors has been the object of several studies during the last few decades. Non-coding genetic material, in particular miRNAs and lncRNAs, can modulate genic expression and interfere at a transcriptional level with some pathways that are well known to be crucial in the pathogenesis of cancers, potentially acting as oncogenes or onco-suppressors.

These molecules have acquired a relevant role as potential diagnostic, prognostic, or predictive markers, in the diagnostic definition and management of patients.

Albeit a rare cancer, there is growing evidence suggesting the possible advantages of using non-coding genetic material as helpful markers in ACC.

As such, both circulating and tissue miRNAs have been described as potential helpful prognostic markers in differentiating benign versus malignant adrenal tumors. Furthermore, the study of some miRNA levels might be able to predict the response to mitotane and doxorubicin or streptozocin treatment. However, despite advances, further studies are required to define the role of miRNAs as markers of prediction of response to treatments in adrenal tumors.

The dysregulation of some miRNAs in patients with ACC has also been related to tumoral hormonal secretion, suggesting a potential role of miRNAs in the early diagnosis and in the follow-up of subclinical endocrine disorders due to hormone secretion. In any case, whether there is a correlation between miRNAs levels and the hormonal excess severity remains to be further clarified. Therefore, future studies are required to define the potential role of non-coding genetic material as early diagnostic or prognostic markers of long-term complications in clinical disorders associated with adrenal hormonal dysfunctions.

Recently, in vitro and in vivo studies have investigated the pathogenic role of miRNAs in preclinical models of ACC, including human ACC cell lines. These studies suggest that some miRNAs might play a role in the pathogenesis targeting signaling pathways that are involved in cellular growth and proliferation, such as IGF/Akt/mTOR, or proteins involved in apoptosis as Bcl2 and PAX. Differently, other miRNAs have been found to affect some transcription factors, such as DICER and TARBP2, with a role in tumorigenesis that is still unclear.

In contrast, only a small number of studies exists on the role of lncRNAs in ACC when compared with the number of articles reporting miRNAs. Despite this lower level of knowledge, some lncRNAs have also been suggested to be potential diagnostic and prognostic markers in ACC. To date, different potential carcinogenic lncRNAs have been identified and may play a role in the pathogenesis of ACC as in other tumor types.

In conclusion, non-coding genetic material, in particular miRNAs and lncRNAs, might play a role as diagnostic or prognostic markers or predictors of response to treatment in patients with adrenocortical tumors becoming potential beneficial markers for the diagnostic and therapeutic stratification of patients. However, further studies are still required to be able to apply this knowledge in the routine management of patients.

## Figures and Tables

**Table 1:** Circulating miRNA in adrenocortical tumors.

Tumor Entity	miRNA	Expression
Adrenocortical carcinoma	miR-483-5p	Overexpression vs. A
	miR-101	Overexpression vs. A
	miR-100	Overexpression vs. A
	miR-181b	Overexpression vs. A
	miR-184	Overexpression vs. A
	miR-210	Overexpression vs. A
	miR-195	Underexpression vs. A
	miR-335	Underexpression vs. A
Primary hypercortisolism	miR-22-3p	Overexpression vs. NA
	miR-27a-3p	Overexpression vs. NA
	miR-320b	Overexpression vs. NA
	miR182-5p *	Overexpression vs. healthy
	miR133a-3p *	Overexpression vs. healthy
	miR200b-3p **	Overexpression vs. healthy
	miR-449 ***	Overexpression vs. CPA
	miR-142 ****	Overexpression related to ARMC5 wild type
Primary hyperaldosteronism ***	miR-30d-5p	Overexpression in hyperplasia vs. APA
	miR-7-5p	Overexpression in hyperplasia vs. APA
	miR-30e-5p	Overexpression in hyperplasia vs. APA

A = adrenocortical adenoma; NA = non secreting adenoma; CPA = cortisol producing adenoma; APA: aldosterone producing adenoma; vs. = versus; * in the context of Cushing’s disease ** myomirs; *** in the context of primary pigmented nodular adrenocortical disease; *** main evidences in hyperaldosteronism due to bilateral adrenal hyperplasia; **** in the context of primary macronodular adrenal hyperplasia.

**Table 2:** Tissue miRNA in adrenocortical tumors.

Tumor Entity	miRNA	Expression
Adrenocortical carcinoma	miR-483-5p	Overexpression A
	miR-184	Overexpression N, A
	miR-503	Overexpression N, A
	miR-508-3	Overexpression N A
	miR-210	Overexpression A, N
	miR-483-3p	Overexpression A
	miR-335	Underexpression N, A
	miR195	Underexpression A, N
	miR-511	Underexpression A, N
	miR-214	Underexpression A, N
	miR-497	Underexpression A, N
	miR-7	Underexpression N, A
Primary hypercortisolism	miR-210	Underexpression vs. ACC
	miR-375	Underexpression vs. N
Primary hyperaldosteronism *	miR-23 *	Overexpression related to higher aldosterone secretion
	miR-34a *	Overexpression related to higher aldosterone secretion
	miR-320a-3p	Overexpression vs. N
	miR-193a-3p	Underexpression vs. N
	miR-125a-5p	Underexpression vs. N
	miR-495-3p	Underexpression vs. N

Over/underexpression of miRs in ACC compared to: A = adrenocortical adenoma; N = normal adrenal gland; APA: aldosterone producing adenoma; vs. = versus; * in the context of APA.
